# Correction: Banack, S.A. *et al*. Production of the Neurotoxin BMAA by a Marine Cyanobacterium. *Mar. Drugs* 2007, *5*, 180–196

**DOI:** 10.3390/md8072013

**Published:** 2010-06-29

**Authors:** Sandra Anne Banack, Paul Alan Cox

**Affiliations:** Institute for Ethnomedicine, Jackson, WY 83001, USA; E-Mail: sandra@ethnomedicine.org

We found an error in our paper published in Marine Drugs [1], in Figure 1, on page 181. The figure showed an incorrect structure for BMAA. A correct structure is provided here (Figure 1). The conclusions of the article remain unchanged.

## Figures and Tables

**Figure 1:**
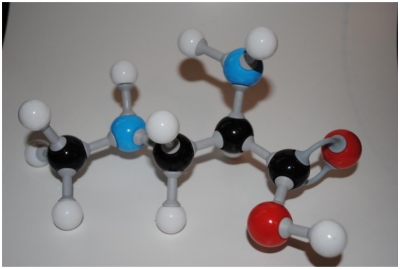
BMAA.
